# Author Correction: Metabolic modeling reveals a multi-level deregulation of host-microbiome metabolic networks in IBD

**DOI:** 10.1038/s41467-025-64877-y

**Published:** 2025-10-09

**Authors:** Jan Taubenheim, A. Samer Kadibalban, Johannes Zimmermann, Claudia Taubenheim, Florian Tran, Stefan Schreiber, Philip Rosenstiel, Konrad Aden, Christoph Kaleta

**Affiliations:** 1 Research Group Medical Systems Biology, Institute of Experimental Medicine, Kiel University and University Hospital Schleswig-Holstein, Kiel, Germany; 2 Research Group Evolutionary Ecology and Genetics, Zoological Institute, Kiel University, Kiel, Germany; 3 Clinic for Internal Medicine II, Hematology and Oncology, University Hospital Schleswig-Holstein, Kiel, Germany; 4 Institute of Clinical Molecular Biology, Kiel University and University Hospital Schleswig-Holstein, Kiel, Germany; 5 Department of Internal Medicine I, University Hospital Schleswig-Holstein, Kiel, Germany; 6 Present Address: Institute of Clinical Chemistry, University Hospital Schleswig-Holstein, Kiel, Germany; 7 Present Address: Cluster of Excellence Balance of the Microverse, Friedrich Schiller University, Jena, Germany

**Keywords:** Computer modelling, Metagenomics, Inflammatory bowel disease

Correction to: *Nature Communications* 10.1038/s41467-025-60233-2, published online 2 June 2025

In the version of the article initially published, Stefan Schreiber’s name was missing from the author list and “Author contributions” and has now been added. Additionally, the “Competing interests” statement incorrectly read “The authors declare no competing interests” and has now been amended to list the relevant interests. These corrections have been made to the HTML and PDF versions of the article.

