# Supplementary material for: Metabolic modeling reveals a multi-level deregulation of host-microbiome metabolic networks in IBD
Source: Nat Commun. 2025 Jun 2;16:5120. doi: 10.1038/s41467-025-60233-2 (PMC12130198; doi:10.1038/s41467-025-60233-2)
Supplement: Supplementary file 1 — Supplementary Information [file 41467_2025_60233_MOESM1_ESM.pdf]

Supplementary information

Description of the general network topology

We based the network reconstruction on significantly associated reactions with the tested conditions in IBD patients, separated by tissue (blood and gut biopsy). Reactions formed the edges, while their substrates were displayed as nodes. The network topology was characterized using the log of edge centrality as a measure of distance between the nodes (Supplementary Fig. 3).

The analysis revealed two major points: Firstly, the biopsy network topology was largely unstructured, comprising a single large network without distinct subnetworks (Supplementary Fig. 5A), except for a small cluster involving maltose metabolism (Supplementary Fig. 5B). In contrast, the blood network had a central main subnetwork and three additional subnetworks. Each of these subnetworks contained at least one central metabolite, indicating their respective function – one involved D-galactose and carbohydrate metabolism. Another was associated with lipid metabolism (containing cholesterol, cholesterol-ester, phosphatidylcholine, and phosphatidylethanolamine). The last subnetwork contained reactions for amino acid metabolism indicated by the presence of arginine, tryptophan, lysine and histidine in the network (Supplementary Fig. 5B). Secondly, the hub metabolites (Supplementary Fig. 5C) were centrally located within the networks/subnetworks, underlining their importance in the inflammation-induced changes in host metabolic activity. Notably, the cofactors NAD/NADP(H) were positioned at the center of both networks, emphasizing their significance in inflammation-related host metabolism.

To understand the positioning of hub metabolites (compare Figure 2B) as well as microbial metabolites, and those derived from metabolomic data within the network topologies, we highlighted the nodes and their connecting reactions (Supplementary Fig. 5C, D).

Detailed description of modeling results for remission and responding patients

For the microbial changes, we observed reduced NAD (de-)phosphorylation (Supplementary Fig. 14B) and increased production of 3-dehydrocholate (Supplementary Fig. 14D) in responding patients. The former fits to the observation of more de-novo NAD synthesis during inflammation, while the latter indicates more active de-conjugation of bile acids. Surprisingly, we observed a reduced activity of the mixed acid fermentation pathway for responders (Supplementary Fig. 14B), which predominantly produces lactate, acetate, and formate, but not butyrate or propionate. This correlates to the increased exchange of lactate, decreased microbial exchange of propionate, and reduced host-relevant butyrate production during inflammation (Supplementary Fig. 14B, Figure 1C, D).

For remission, we observed an increase in pyrimidine salvage and nucleotide synthesis (Supplementary Fig. 14F – both pathways necessary for nucleotide and NAD synthesis (reduced during inflammation, Figure 1B). Nicotinamide was also less exchanged in responder microbiomes and more available for the host (Supplementary Fig. 14G, H). For amino acids, we found reduced synthesis of lysine, leucine, and arginine (Supplementary Fig. 14F), resulting in less arginine (and glutamine) cross-feeding between

bacteria (Supplementary Fig. 14G). Concurrently, we observed increased consumption of dietary proline with treatment response (Supplementary Fig. 14H). This contrasts with the increased host availability of leucine, asparagine, and proline during inflammation (Figure 1D). Hence, competition for proline seems crucial for healthy microbiome-host interactions.

Further, we observed increased production of monosaccharides (xylitol, glucose) and their fermentation products (malate), while complex carbohydrates (starch, malto-11-ose) were less available to the host (Supplementary Fig. 14H). This suggests an enhanced degradation of resistant carbohydrates and their usage in fermentation processes in responder microbiomes. Homocysteine-methionine-cysteine interconversion was reduced in the response phenotype (Supplementary Fig. 14F), indicating an increased availability of these compounds, including homocysteine, which was identified as central to the metabolic changes during inflammation in the host (Figure 6G, H). Additionally, we observed a decrease in synthesis of fatty acids, teichoic acid, phosphatidylethanolamines, and farnesol (Supplementary Fig. 14F) which could result in reduced usage of SCFAs in these pathways and increased availability for the host. Furthermore, we identified increased β-alanine production as positively associated with remission. Beta-alanine is produced from propionate and is used to produce coenzyme A, thereby important for lipid metabolism (Supplementary Fig. 14F). This relates back to the inflammation-induced changes in the microbial metabolism, where we observed decreased propionate exchange among bacteria, thus less usage of propionate in their own metabolic processes. We also found decreased synthesis of lipoteichoic acid and arachidonoylglycerol, indicating a reduced demand for coenzyme A (Figure 1B, C).

For the changes in host metabolism, we found increased amino acid metabolism (arginine, proline, glycine, serine, alanine, threonine) and increased glutathione metabolism in patients responding to treatment (Supplementary Fig. 16C), reversing some effects observed during inflammation. In patients undergoing remission, we observed increased sphingolipid metabolism in biopsies, which were reduced during active disease in blood (Supplementary Fig. 16G). Hence, the gut seems to compensate for some of the changes observed in blood during remission.

When analyzing metabolite enrichment in the significantly associated reactions with response and remission, we found no consistent patterns between blood and gut tissue (Supplementary Fig. 16D, H). Notably, for response, the enrichment of NAD(H) in biopsies and coenzyme A in blood was driven by more upregulated reactions in responsive patients, contrasting with the downregulation of these metabolites during inflammation (Supplementary Fig. 16D).

In the analysis of the metabolomics results associated with response and remission, we observed an increase of serum levels of lysophosphatidylcholines, phosphatidylcholines, and choline (response only) (Supplementary Fig. 13A, D), contrary to the decreased levels observed during inflammation (Figure 3A). This further indicates increased availability of choline for recycling homocysteine to methionine in patients responding to the treatment (Figure 6G, H). Relatedly, we found increased levels of cystine (cys-S-S-cys) in the same analysis, another indicator of sufficient homocysteine levels and a substrate for glutathione production (Supplementary Fig. 13A, Figure 6E-H). In addition, we identified higher levels of kynurenine and serotonin in treatment-responsive patients (Supplementary Fig. 13A), while we found no association with tryptophan, indicating sufficient levels of tryptophan to fuel de-novo NAD synthesis (Figure 6A-D) and even production of serotonin from it.

Finally, we repeated the network reconstruction to find interconnections between microbial, host, and blood metabolomics metabolites for patients in response and remission. Due to the

fewer reactions identified in the host metabolism under these conditions, we recovered smaller networks for all conditions (Supplementary Fig. 18, Supplementary Data 20, Supplementary Data 21, Supplementary Data 22, Supplementary Data 23). Especially for remitting patients, we recovered networks too small to establish any direct interactions between the different metabolites (Supplementary Fig. 18E-H). For response, we again found significantly shorter paths between center, microbial, and metabolomic metabolites than between all other metabolites in the network (Supplementary Fig. 19C). Nonetheless, in both tissues, we found similar connections between the choline to phosphatidylcholines (Cen-Met) and phosphatidylcholines to homocysteine (Mic-Met) as in the inflammation network (Supplementary Fig. 19B, D).

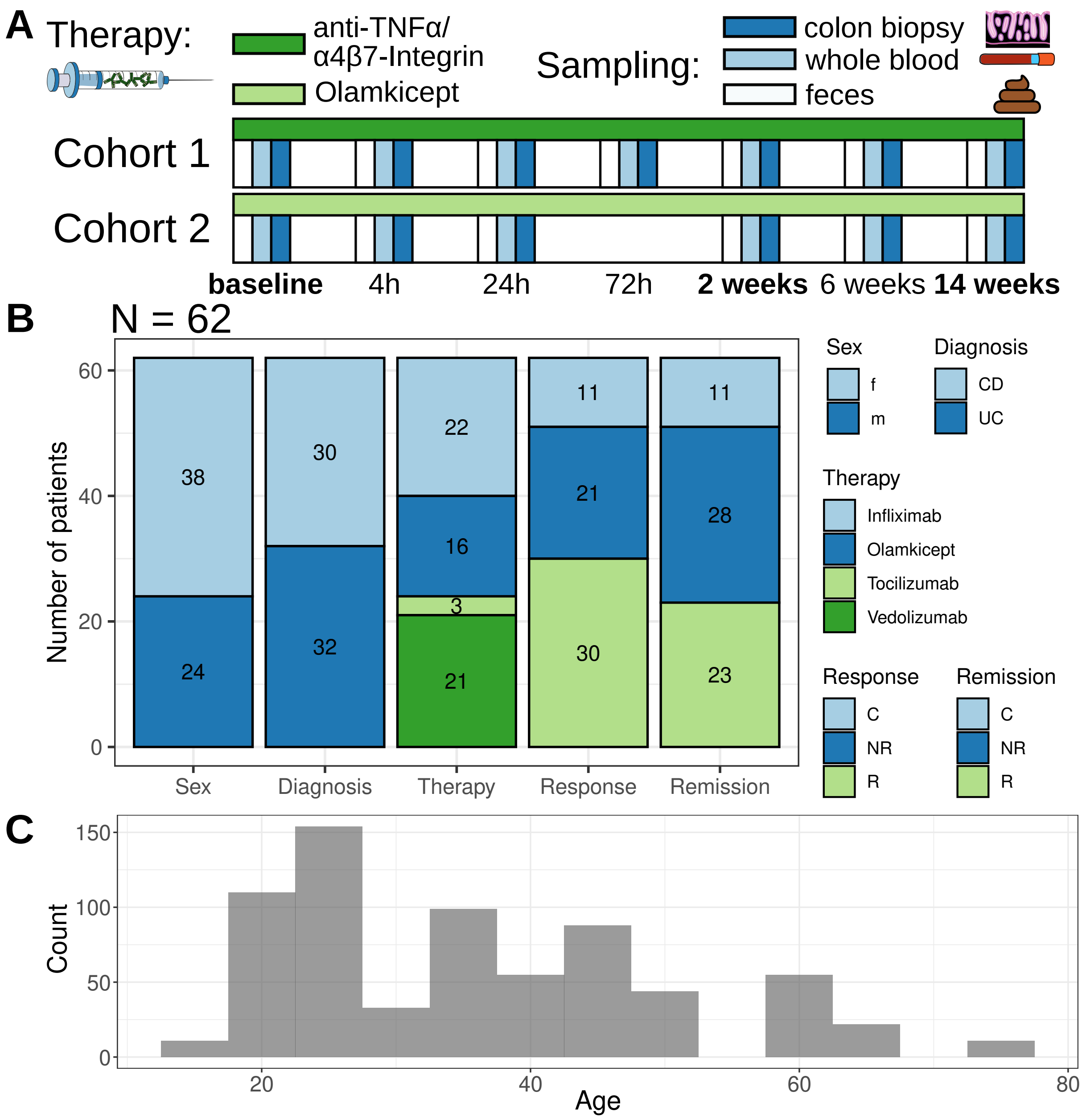

Supplementary Fig. 1: Cohort characteristics (1,2). (A) In the cohort treatment plan and sampling strategy, bold font indicates sampling of the majority of the patients in the cohort. (B) General patient statistics and (C) patient age distribution are displayed. Abbreviations: f - female, m - male, C - control (non-inflamed IBD patients), NR - non-responder/non-remitter, R - responder/remitter

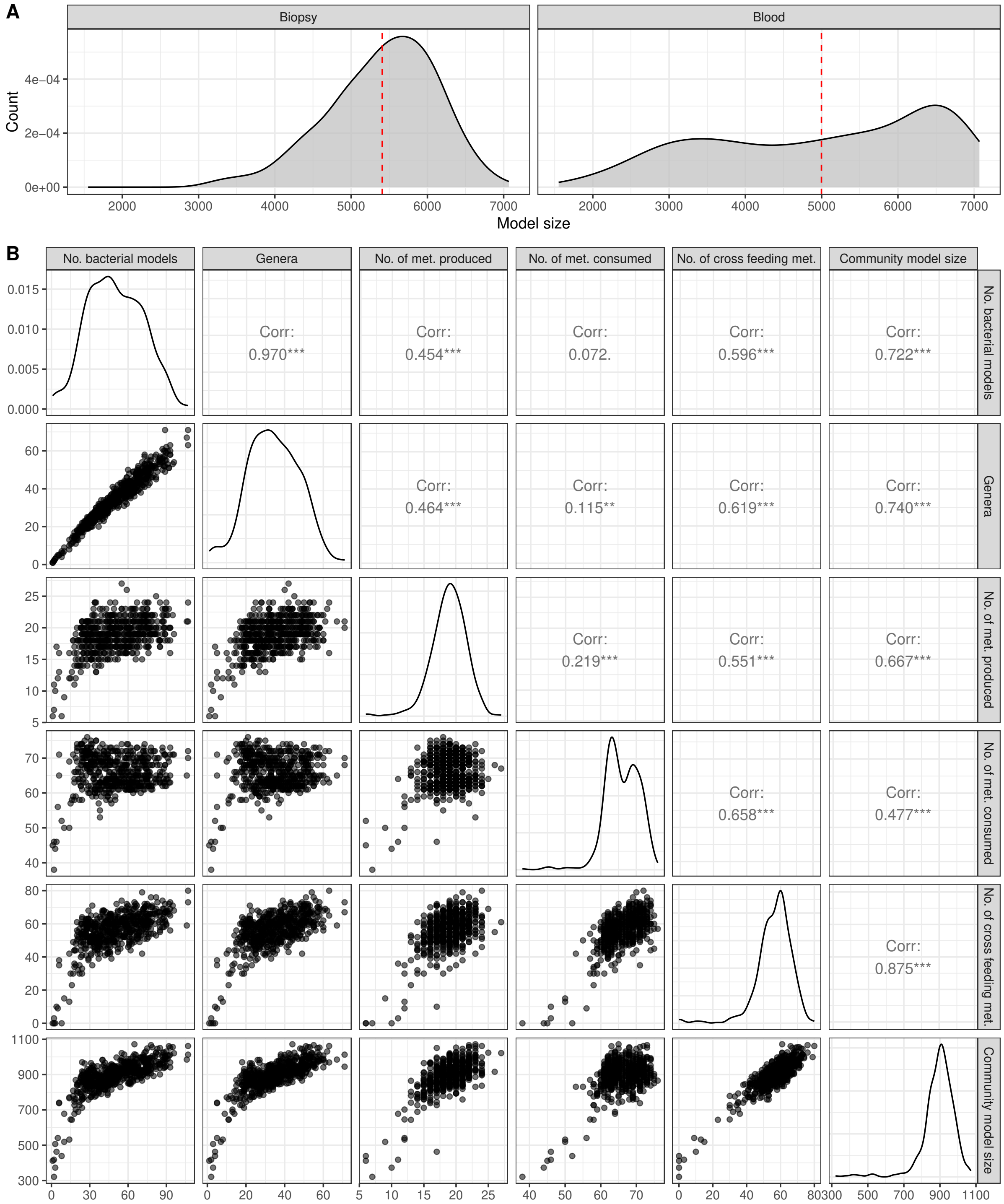

Supplementary Fig. 2: General statistics of reconstructed metabolic models. (A) Distribution of model sizes for reconstructed context specific metabolic models for biopsy and blood samples. Red lines indicate mean model size. (B) Distribution and correlation of key statistics of the reconstructed community models for the microbiome of IBD patients.

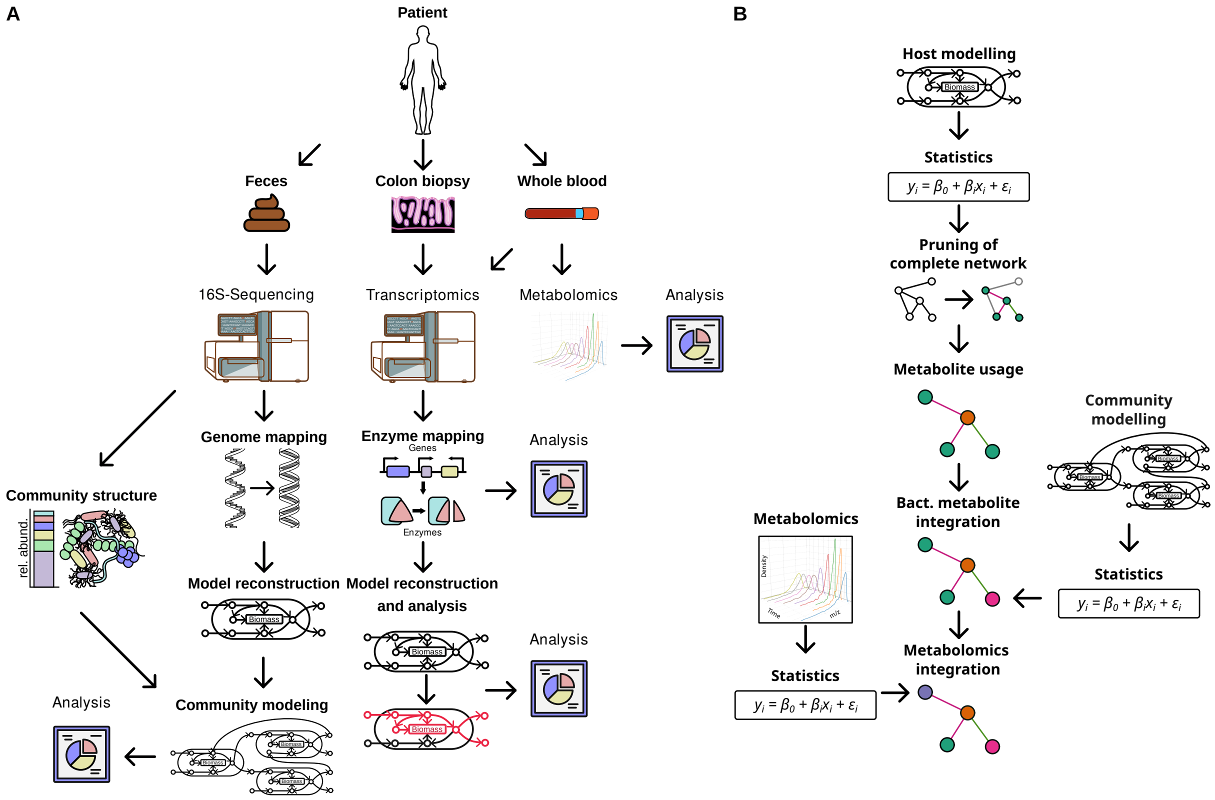

Supplementary Fig. 3: Flow chart describing the data analysis pipelines for (A) the steps from sampling to metabolic modeling and (B) the creation of data integration into disease specific networks and their visualizations in Fig. 4, Supplementary Fig. 5, and Supplementary Data 1-6.

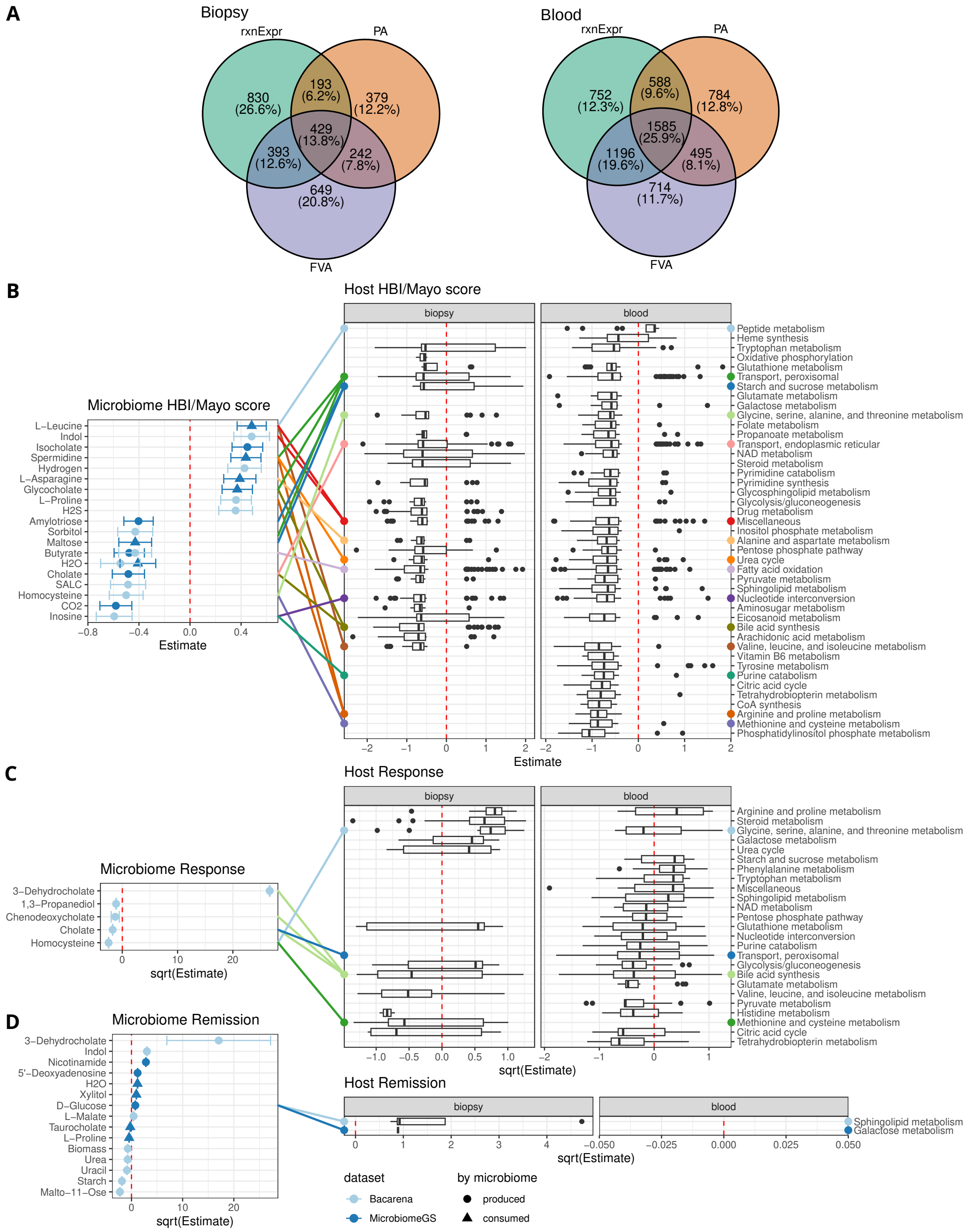

Supplementary Fig. 4: The metabolic changes of the microbiome and the IBD patients which are related to disease activity are cross-linked to each other. (A) Associations of reaction expression (rxnExp), presence/absence (PA) and flux variability analysis results (FVA) to disease activity scores (HBI/Mayo) identified inflammation-dependent reactions in biopsy and blood samples. (B-D) Links indicate connections between microbial metabolites and host subsystems. The analysis shows most changes in the metabolism of host and microbiome which are associated with disease activity (B), second most with response to treatment (C) and only fewer changes in remitting patients (D). n = 565 microbiome samples, n = 296 for biopsies and n = 324 for blood samples, multiple testing adjustments via Benjamini-Hochberg-correction. Abbreviations: reac. - reaction, inflam. - inflammation, SCFA - short chain fatty acids.

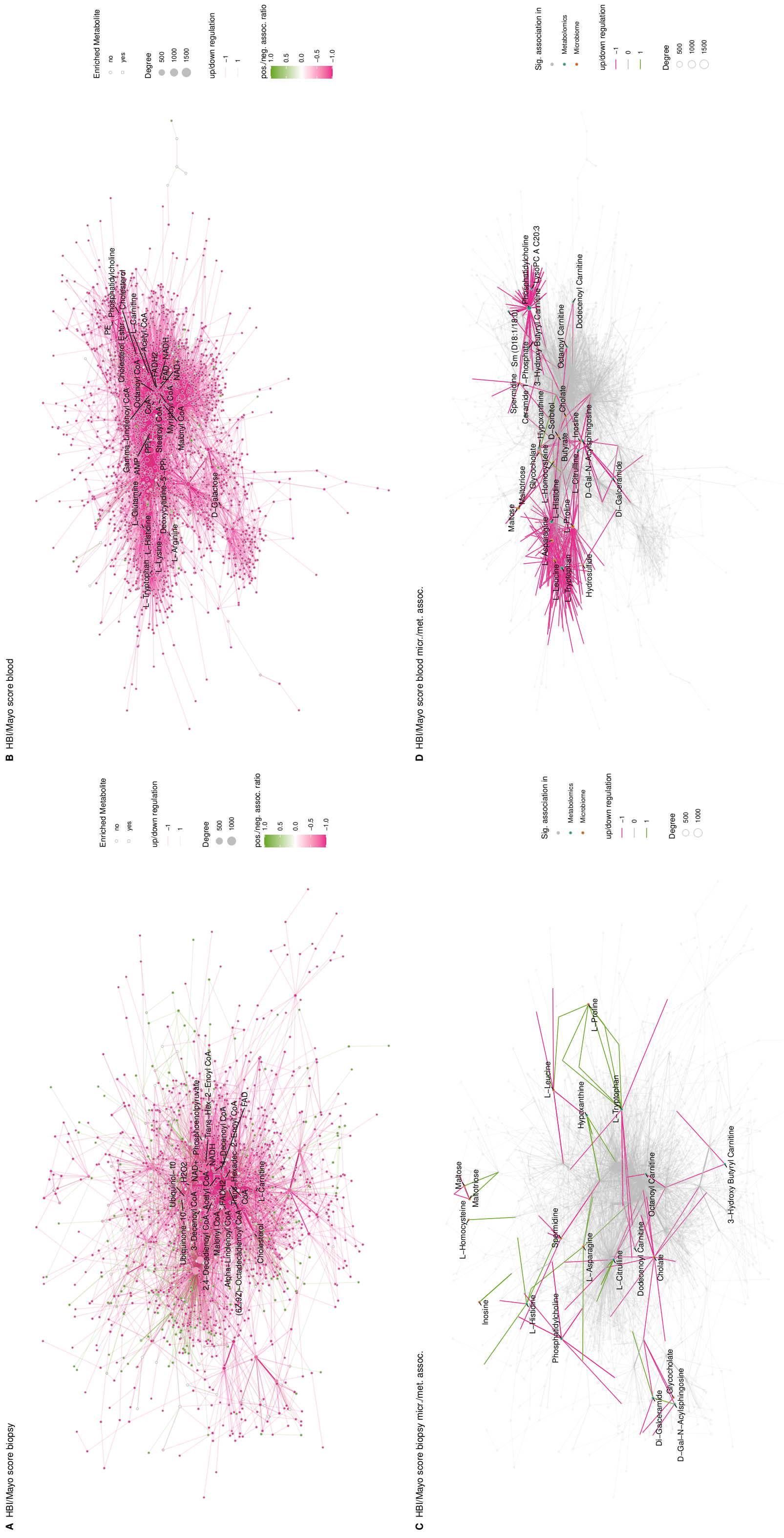

Supplementary Fig. 5: Network representation of the host reactions associated with the change in HBI/Mayo scores for biopsy (A) and blood (B). Metabolites are nodes while the reactions are edges. The topology is based on the node degree and nodes for hub metabolites are labeled (compare Fig. 2 C). (C,D) The same network topology as before, but metabolites involved in host reactions which are either associated to changed microbial production or to changes in the metabolomics data are highlighted and labeled.

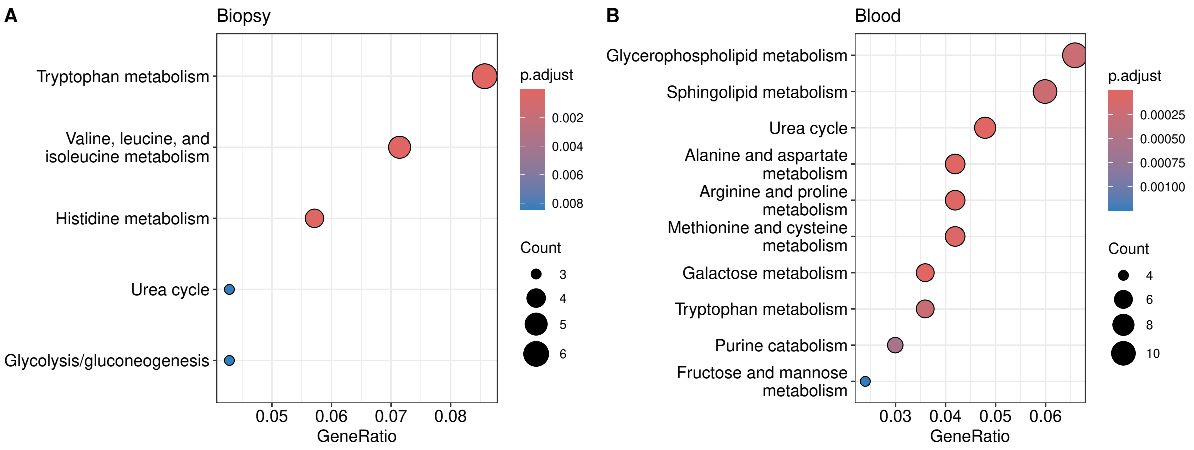

Supplementary Fig. 6 Enrichment analysis for metabolic subsystems with the reactions along the 5% shortest paths of the inflammation specific metabolic network between hub, microbial and metabolomics derived metabolites (compare Fig. 4) for (A) biopsy and (B) blood samples. Hypergeometric tests with Benjamini-Hochberger p-value correction.

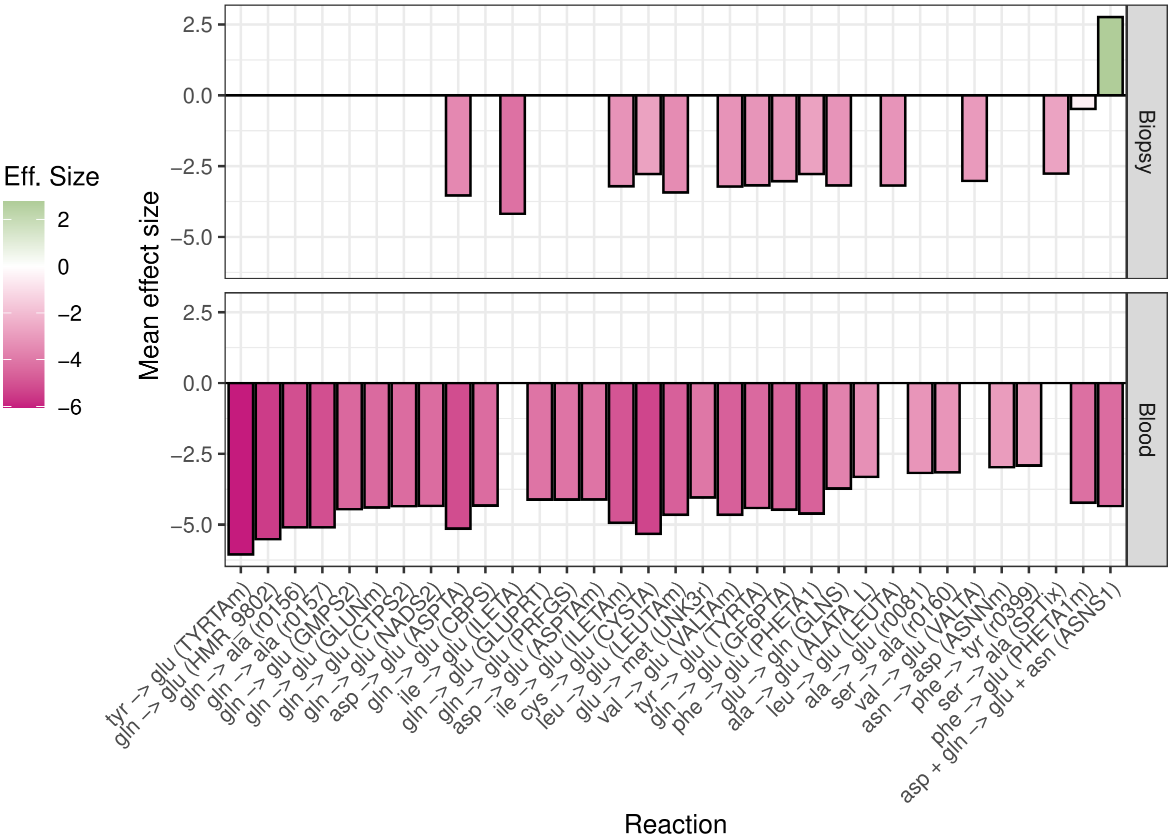

Supplementary Fig. 7: Amino acid transamination reactions are mostly downregulated in biopsy but especially in blood samples.

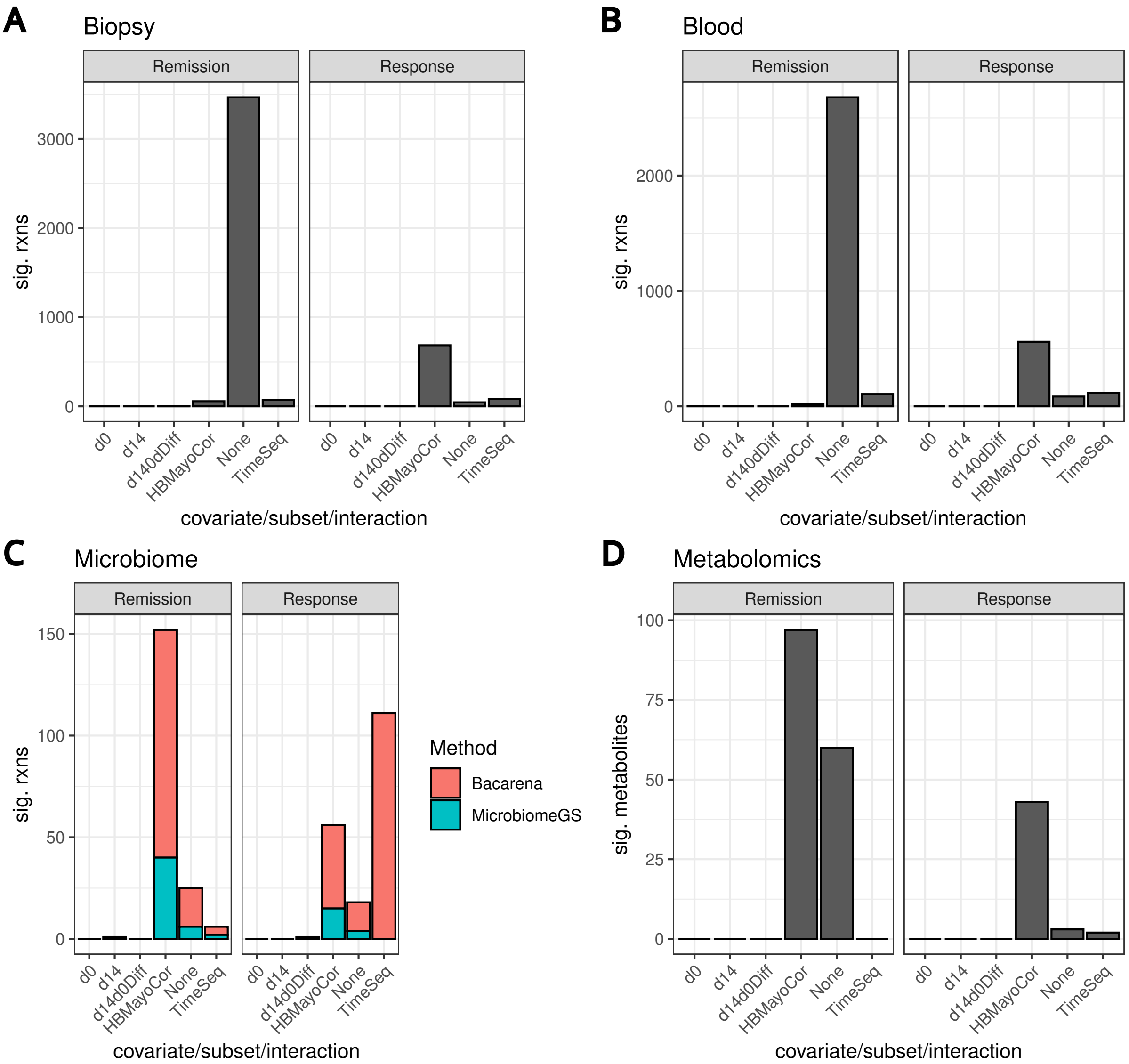

Supplementary Fig. 8: Total number of unique features (reactions/metabolites) which could be significantly associated with remission or treatment response in gut metabolism (A), blood metabolism (B), microbial metabolism (C) and serum metabolite concentrations (D). We used different statistical approaches and data subsets to perform these association: d0 - only baseline samples, d14 - only samples at d14 after treatment, d0d14Diff - change in the feature values between d0 and d14, HBMayoCor - whole dataset with HBI/Mayo score as covariate, None - whole dataset, TimeSeq - testing for changes in remission/responding patients over time.

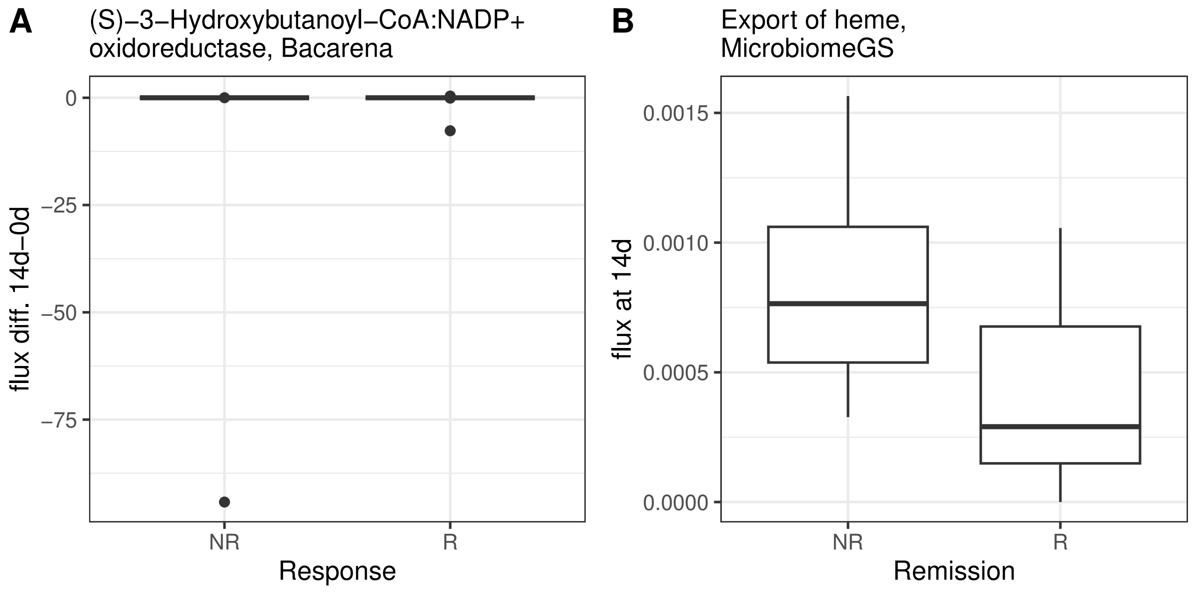

Supplementary Fig. 9: Significant changes in reaction fluxes for microbial metabolism which could serve as a biomarker for remission/response of patients. (A) Changes of fluxes in (S)-3-Hydroxybutanoyl-CoA:NAPD+oxidoreductase between baseline and 14 days after treatment was significantly associated with treatment response, yet the correlation seems spurious. (B) Export of heme by the microbiome was increased 14 days after treatment start for remitting patients. Boxplots: center line, median; box limits, upper and lower quartiles; whiskers, 1.5 times interquartile range; points, outliers. n = 30 response, n = 21 non-response, n = 23 remission, n = 28 non-remission samples. Abbreviation: R - response/ remission, NR - non-response/non-remission.

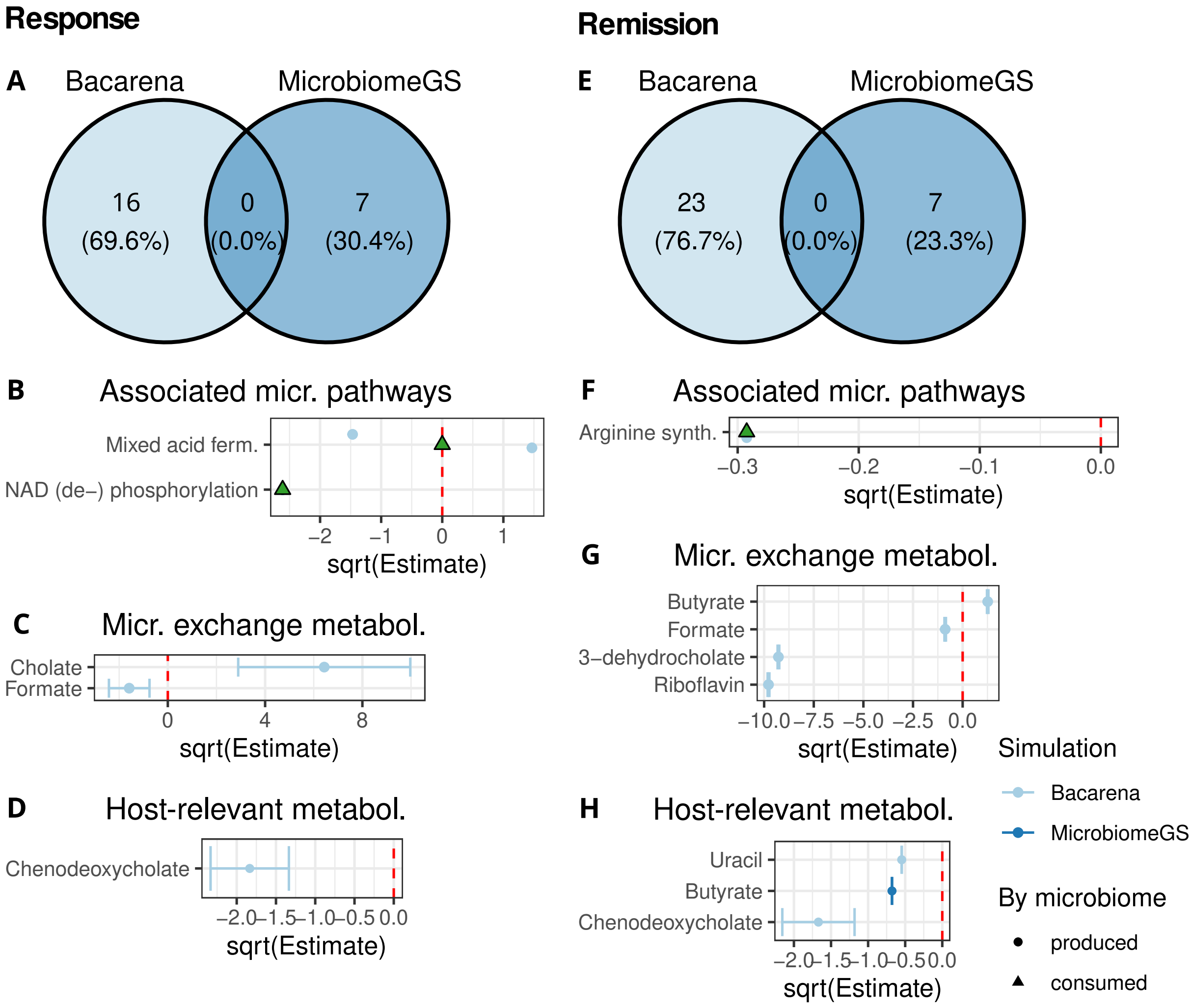

Supplementary Fig. 10: Response (A-D) and remission (E-H) associated changes in microbial community metabolism. Significantly changed reactions from BacArena and MirobiomeGS2 analysis for response (A) and remission (E) are displayed in a Venn diagram. From these reactions we enriched microbial pathways for response (B) and remission (F). Microbiota changed their cross feeding behavior in response (C) and remitting patients (G). Host relevant metabolites have been evaluated in association with response (D) and remission of the patients. The points displayed in B-D and F-H correspond to the estimate for the coefficient of a linear mixed model; error bars represent confidence intervals. Estimates is a measure for the odds ratio of being a responder/remitter, hence values above zero indicate increased metabolic function in remitting/responding patients. Abbreviations: micr. - microbial, reac. - reaction, MGS2 - MicrobiomeGS2, ES - effect size (t-value), n = 565 samples, multiple testing adjustments via Benjamini-Hochberg-correction.

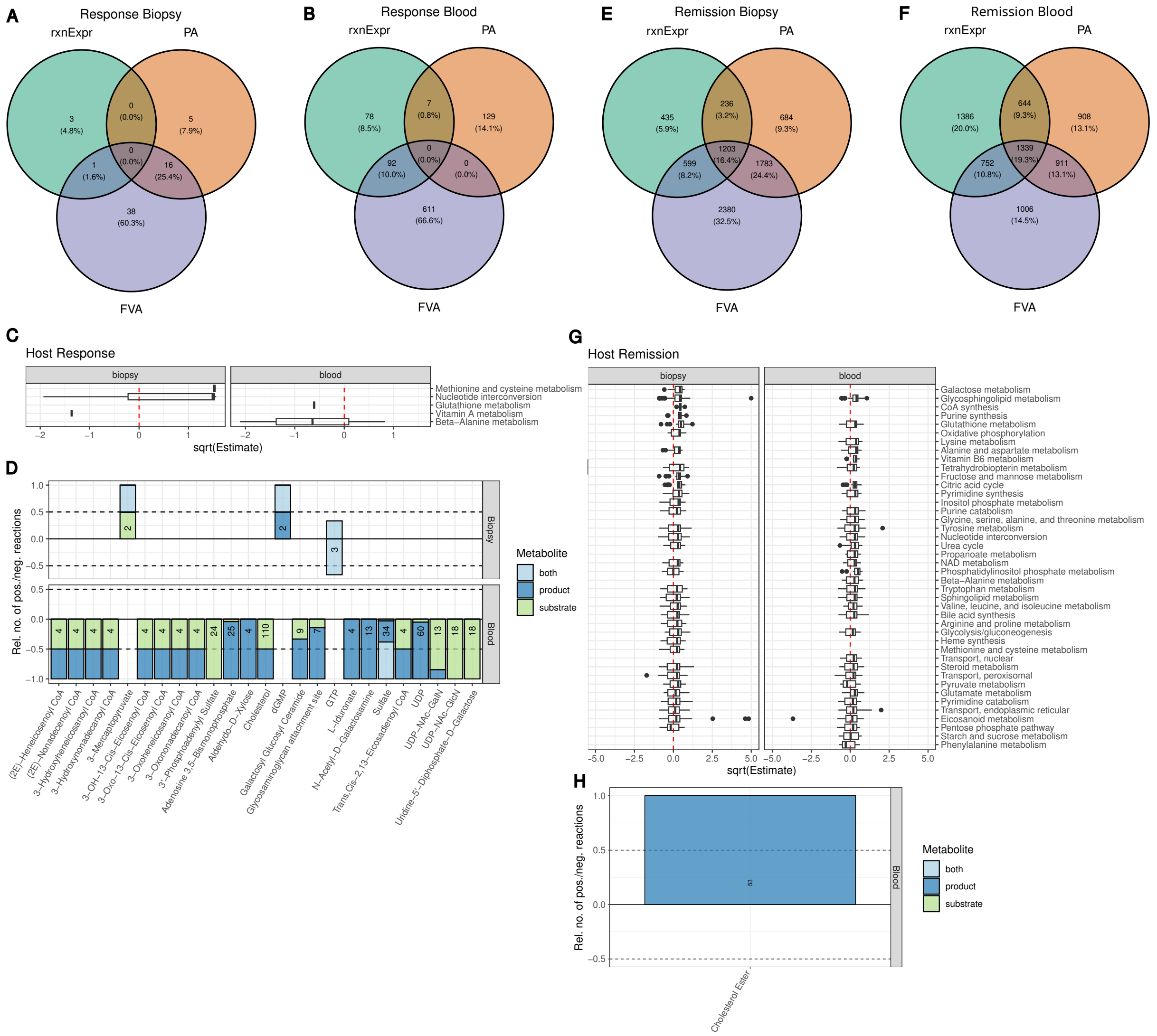

Supplementary Fig. 11: Associations of reaction expression (rxnExp), presence/absence (PA) and flux variability analysis results (FVA) to response (A-B) and remission (E-F) identified relevant reactions in biopsy and blood samples. We enriched subsystems with reactions dependent on response (C) and remission (G) via a set enrichment analysis and a hypergeometric test. The plot shows the estimate of the linear mixed model coefficient for each significantly associated reaction in the subsystem - values above zero indicate increase of reaction activity in response/remission. In a second enrichment analysis, we identified metabolites which are more often used in significantly associated reactions applying a hypergeometric test for response (D) and remission (H) states of the patients. The barplot shows whether the reactions employing the metabolites show in- or decreased activity with the response or remission state (positive and negative values, respectively), while the colors indicate the metabolite function in the respective reactions. Numbers indicate the number of reactions using the metabolite. Boxplots: center line, median; box limits, upper and lower quartiles; whiskers, 1.5 times interquartile range; points, outliers. n = 296 for biopsies and n = 324 for blood samples, multiple testing adjustments via Benjamini-Hochberg-correction. Abbreviations: reac. - reaction, pos. - positive, neg. - negative.

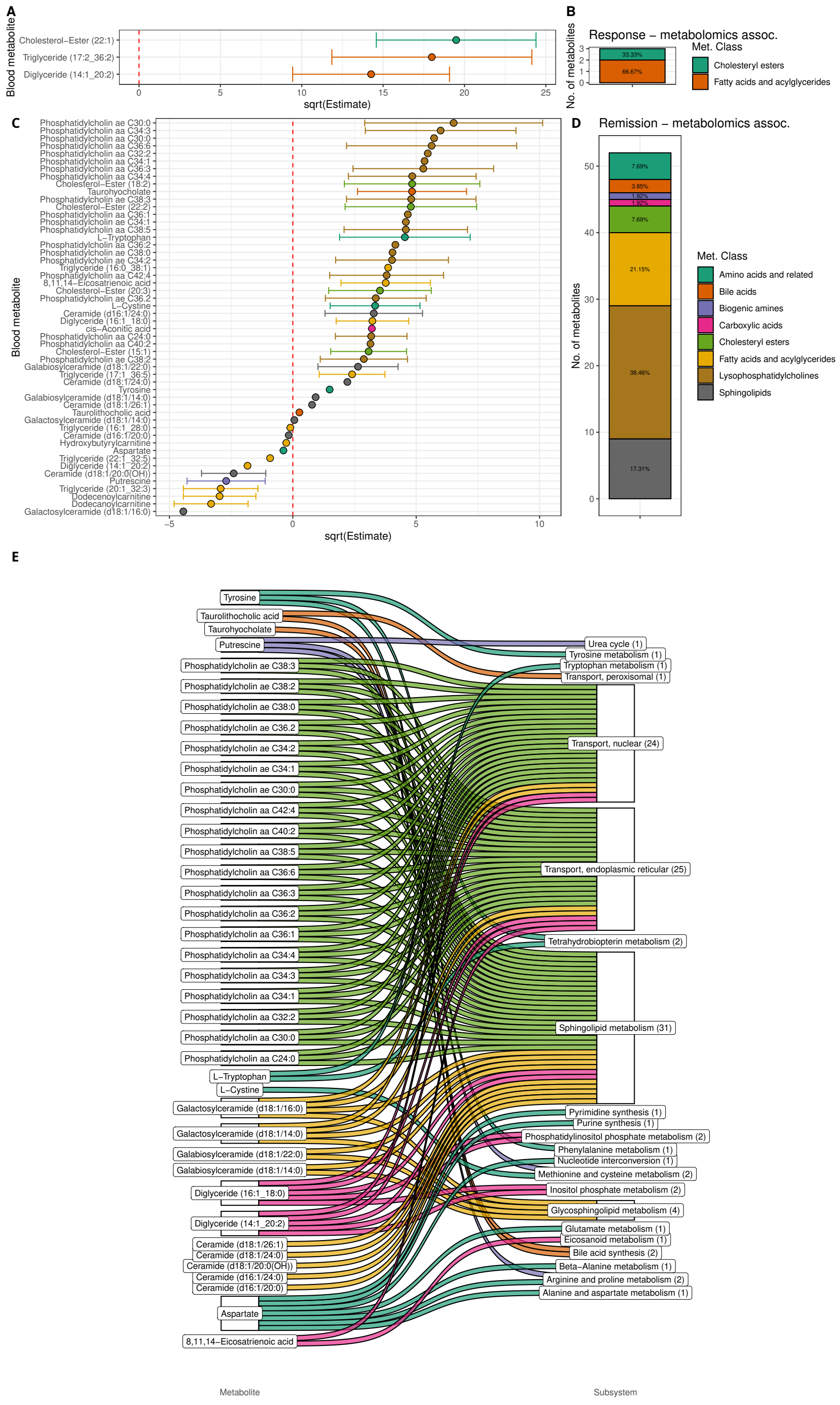

Supplementary Fig. 12: Blood metabolomics show association with treatment response (A) and remission (C) in linear mixed effect models. Dots and error bars represent estimates of the linear mixed model coefficient to response/remission and the respective confidence interval. Values above zero indicate increased metabolite levels are associated with remission/response and vice versa. Amount of metabolites which showed a significant association to either treatment response (B) or remission (D). (E) Metabolites which were associated with remission play a role in the host remission associated subsystems. n = 150 samples, multiple testing adjustments via Benjamini-Hochberg-correction. Abbreviations: assoc. - association, met. - metabolite, no. - number.

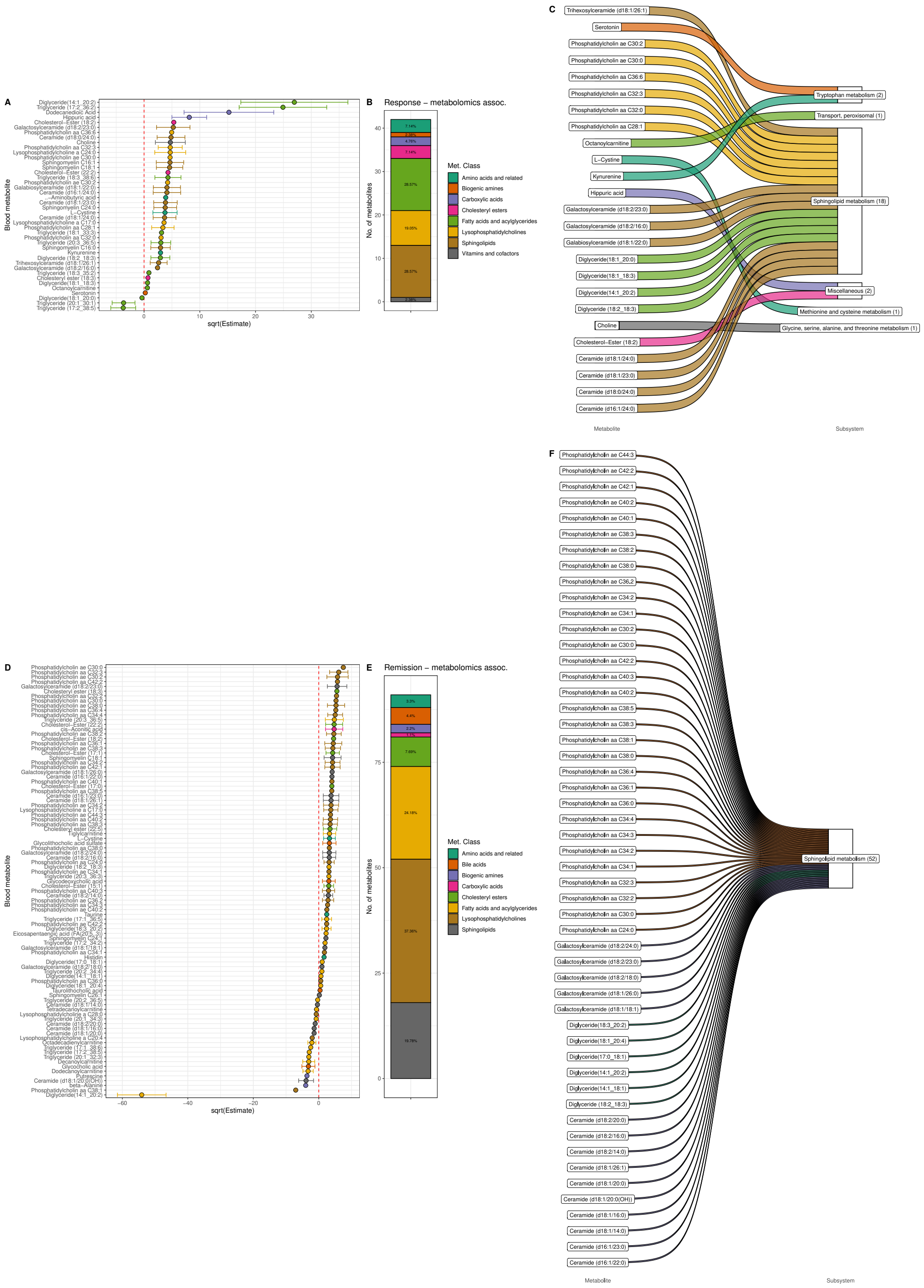

Supplementary Fig. 13: Blood metabolomics show association with treatment response (A) and remission (D) after correction for disease activity in linear mixed effect models. Dots and error bars represent estimates of the linear mixed model coefficient to response/remission and the respective confidence interval. Values above zero indicate increased metabolite levels are associated with remission/response and vice versa. Amount of metabolites which showed a significant association to either treatment response (B) or remission (E). Metabolites which were associated with response (C) or remission (D) play a role in the host remission associated subsystems. n = 150 samples, multiple testing adjustments via Benjamini-Hochberg-correction. Abbreviations: assoc. - association, met. - metabolite, no. - number.

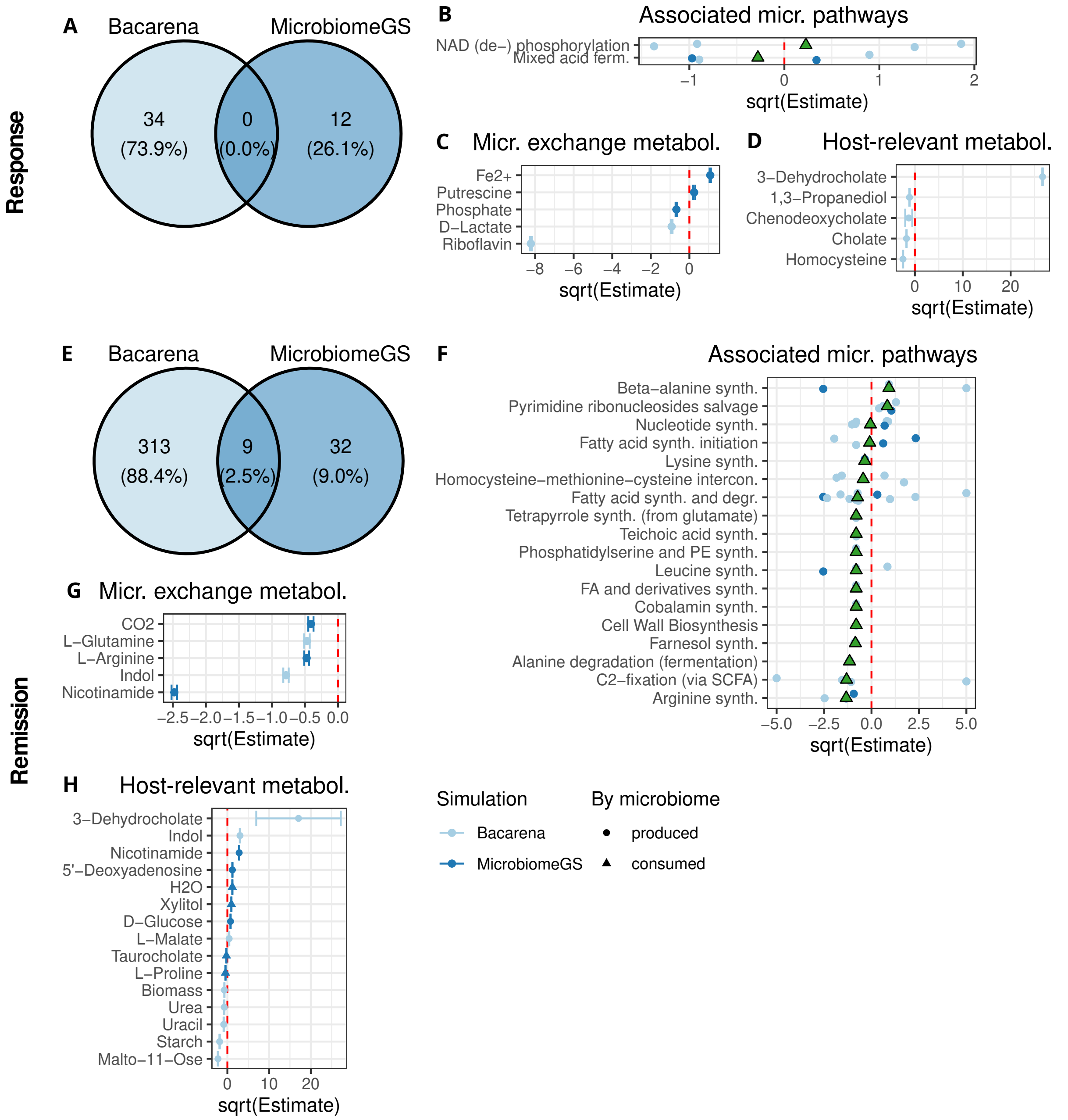

Supplementary Fig. 14: Response (A-D) and remission (E-H) associated changes in microbial community metabolism. Significantly changed reactions from BacArena and MirobiomeGS2 analysis for response (A) and remission (E) with correction for disease activity are displayed in a Venn diagram. From these reactions we enriched microbial pathways for response (B) and remission (F). Microbiota changed their cross feeding behavior in response (C) and remitting patients (G). Host relevant metabolites have been evaluated in association with response (D) and remission of the patients. The points displayed in B-D and F-H correspond to the estimate for the coefficient of a linear mixed model; error bars represent confidence intervals. Estimates is a measure for the odds ratio of being a responder/remitter, hence values above zero indicate increased metabolic function in remitting/responding patients. Abbreviations: micr. - microbial, reac. - reaction, MGS2 - MicrobiomeGS2, ES - effect size (t-value), n = 565 samples, multiple testing adjustments via Benjamini-Hochberg-correction.

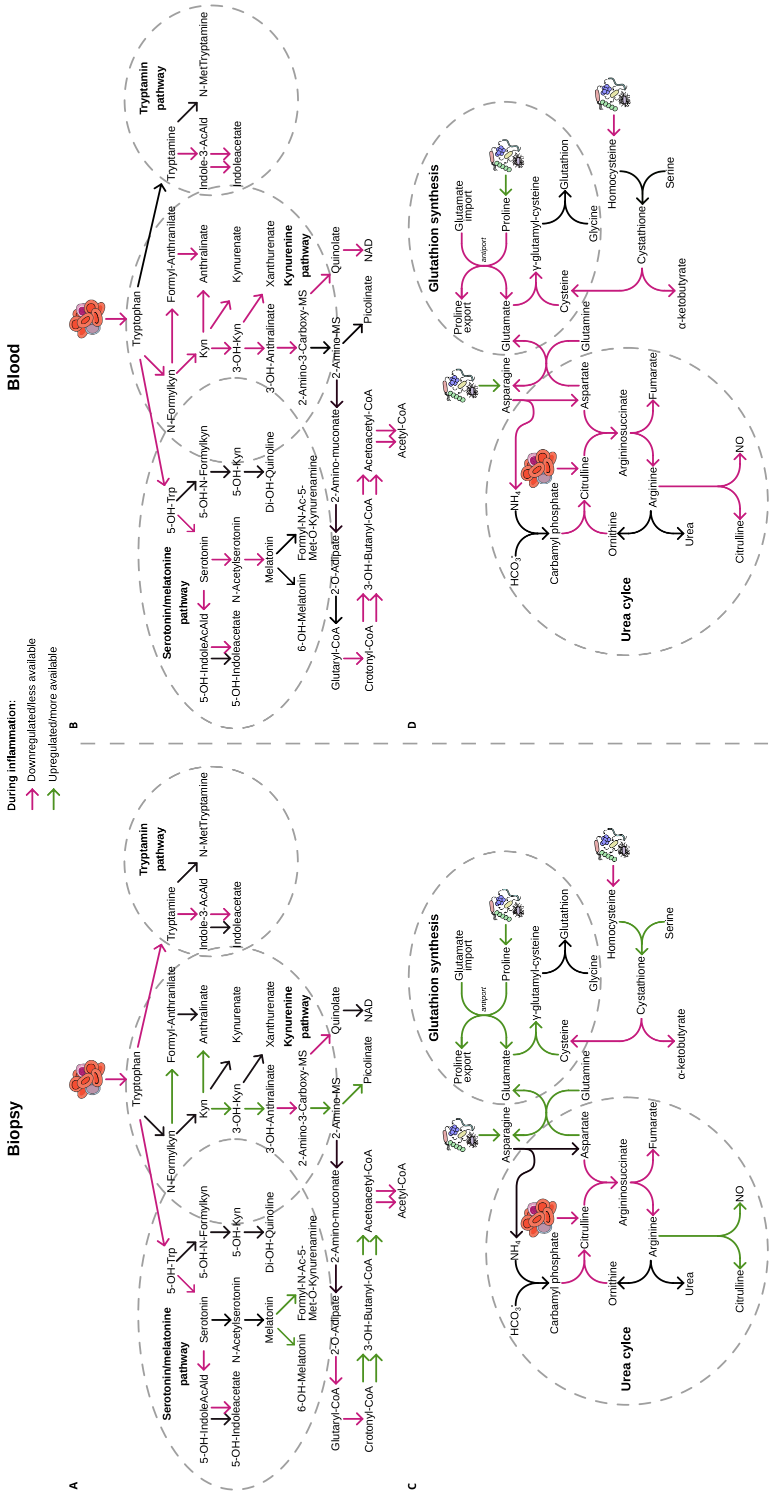

Supplementary Fig. 15: (C, D) Serum tryptophan levels were decreased, while tryptophan degradation via kynurenine was increased in biopsies but not in blood, indicating major loss of tryptophan via the kynurenine pathway in the gut. The production of quinolate in the kynurenine pathway was blocked in both tissues, indicating reduced feed of quinolate to the NAD de novo synthesis pathway. (E, F) The urea cycle and glutathione production are metabolically linked via asparagine synthase, which transforms aspartate and glutamine to asparagine and glutamate. Glutamate, together with cysteine and glycine, forms glutathione - highlighting the pivotal role of glutamate in IBD related metabolism.

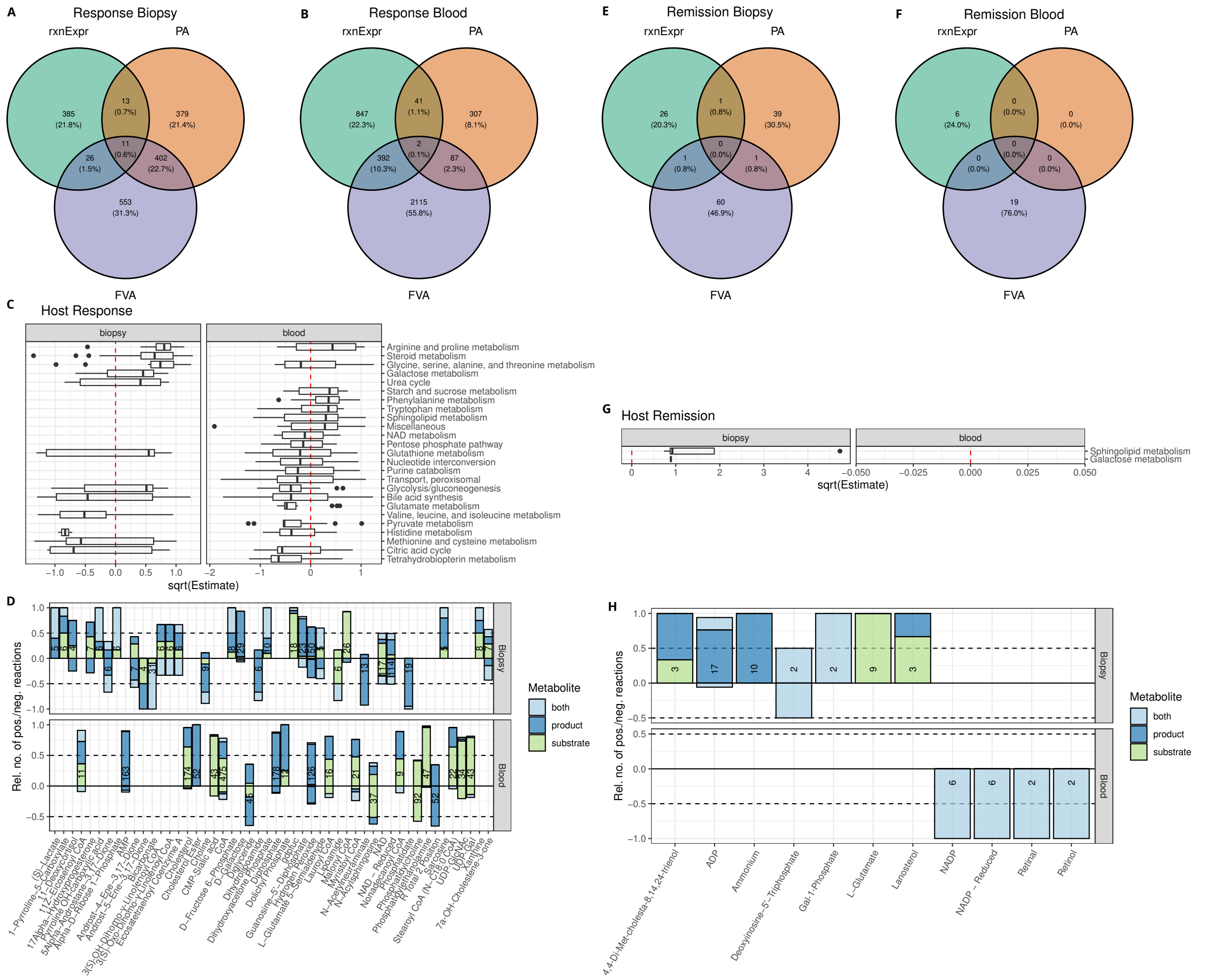

Supplementary Fig. 16: Associations of reaction expression (rxnExp), presence/absence (PA) and flux variability analysis results (FVA) to response (A-B) and remission (E-F) with a correction for disease activity scores identified relevant reactions in biopsy and blood samples. We enriched subsystems with reactions dependent on response (C) and remission (G) via a set enrichment analysis and a hypergeometric test. The plot shows the estimate of the linear mixed model coefficient for each significantly associated reaction in the subsystem - values above zero indicate increase of reaction activity in response/remission. In a second enrichment analysis, we identified metabolites which are more often used in significantly associated reactions applying a hypergeometric test for response (D) and remission (H) states of the patients. The barplot shows whether the reactions employing the metabolites show in- or decreased activity with the response or remission state (positive and negative values, respectively), while the colors indicate the metabolite function in the respective reactions. Numbers indicate the number of reactions using the metabolite. Boxplots: center line, median; box limits, upper and lower quartiles; whiskers, 1.5 times interquartile range; points, outliers. n = 296 for biopsies and n = 324 for blood samples, multiple testing adjustments via Benjamini-Hochberg-correction. Abbreviations: reac. - reaction, pos. - positive, neg. - negative.

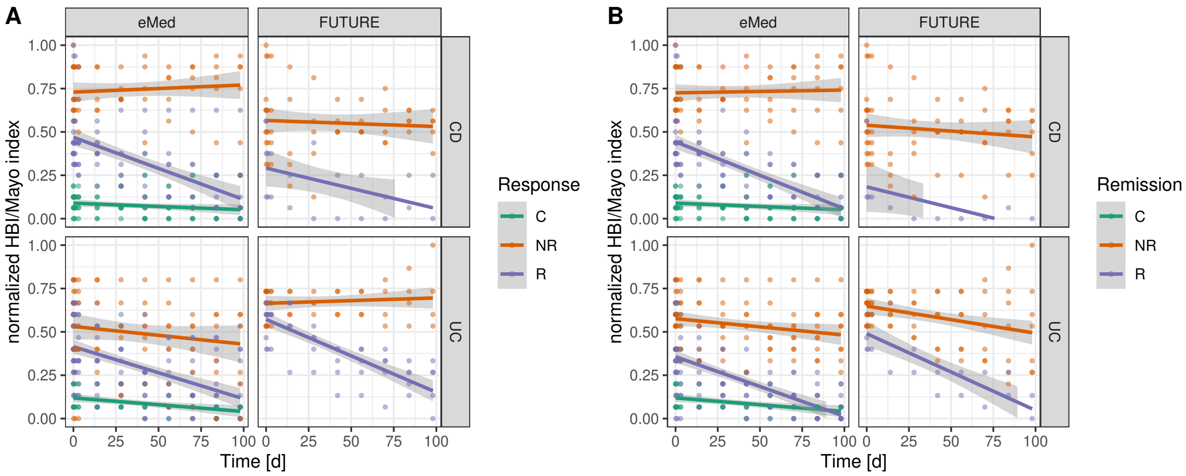

Supplementary Fig. 17: HBI/Mayo score decreased over time for responders (A) and patients going into remission (B), while controls and non-responder/non-remitter keep a relative constant level of inflammation over the time of the study. C - control, NR - non-responder/non-remitter, R - responder/remitter

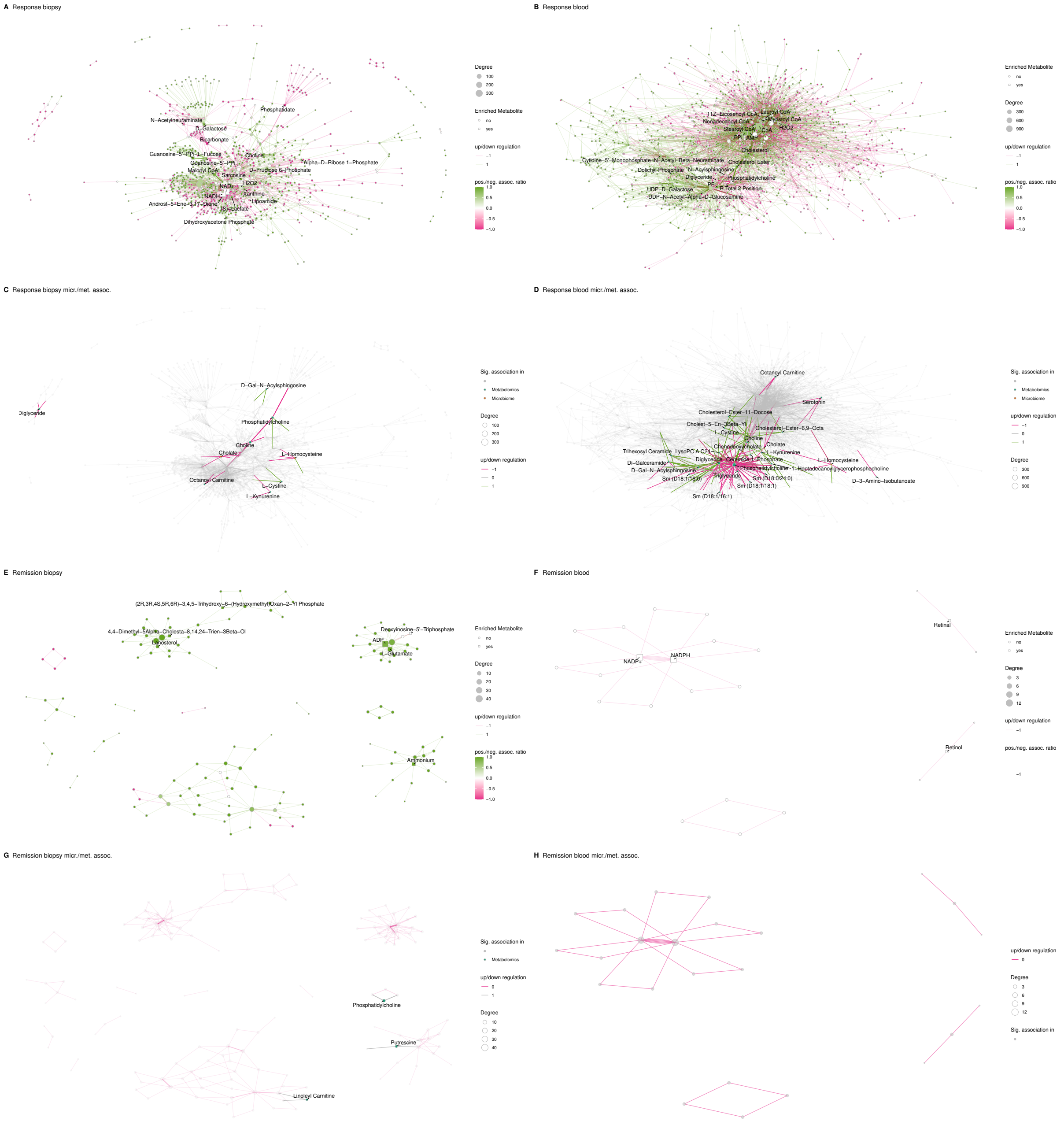

Supplementary Fig. 18: Network representation of the host reactions associated with response to treatment or remission for biopsy (A, E) and blood (B, F), respectively. Metabolites are nodes while the reactions are edges. The topology is based on the node degree (log(sum(degree)) = edge distance). Nodes for center metabolites are labeled (compare Fig. 3 C). (C, D, G, H) The same network topology has been displayed as before, but metabolites involved in host reactions which are either associated to changed microbial production or to changes in the metabolomics data are highlighted and labeled. The networks confirm that center metabolites are central nodes with high degree.

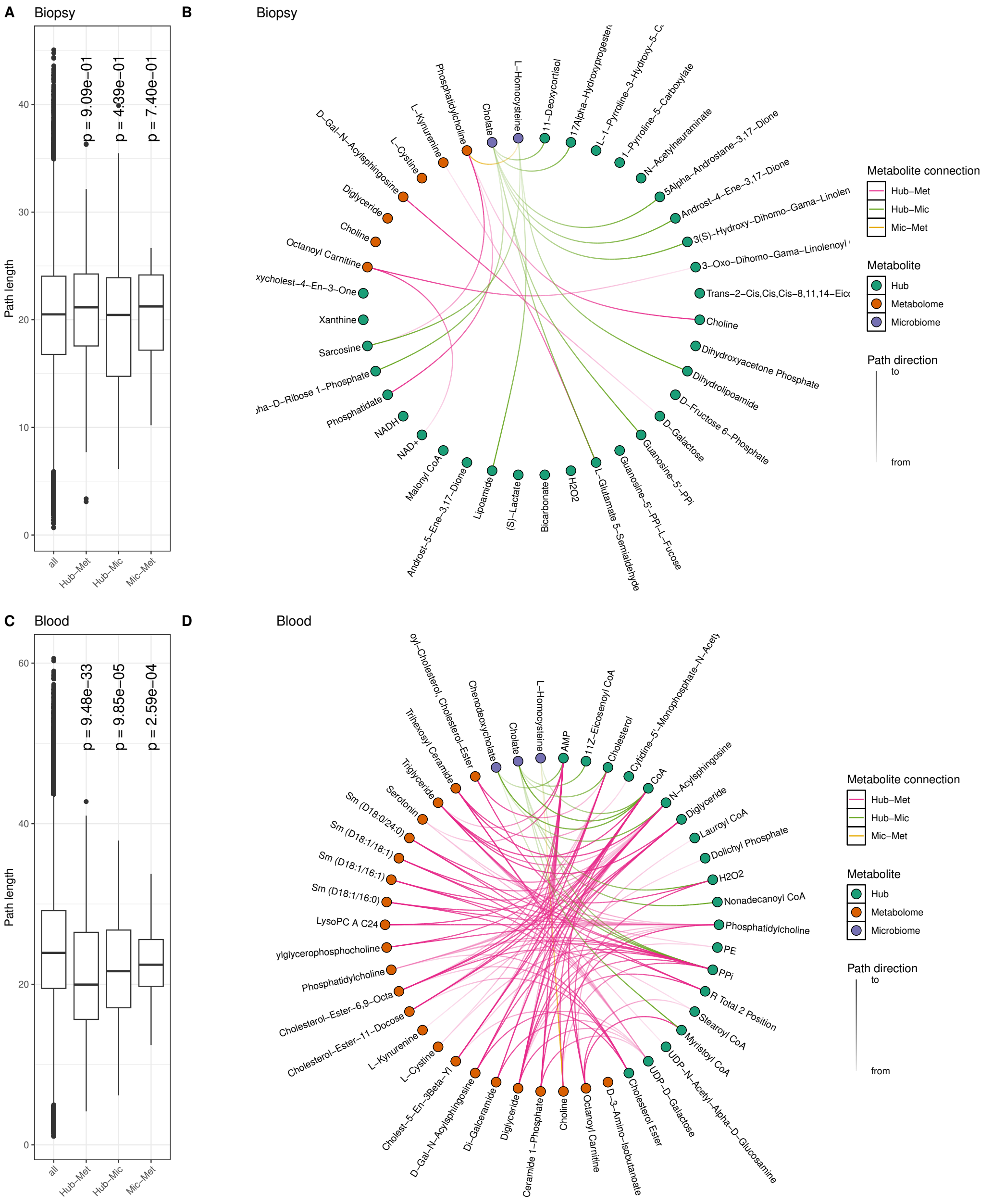

Supplementary Fig. 19: Analysis of network topologies for treatment response. We calculated pathway lengths as the sum of edge lengths between microbial (mic), metabolomics (met) or center (C) metabolites and compared the obtained lengths to the rest of the network distances between all nodes (all) for the biopsy (A) and blood (C) samples. Statistics were obtained by one sided (lesser) student's t-test and FDR correction for multiple testing. Finally we displayed the pathway lengths between the C, mic, and met nodes which belong to the lowest 5% of across the whole network for biopsy (B) and blood samples (D). Boxplots: center line, median; box limits, upper and lower quartiles; whiskers, 1.5 times interquartile range; points, outliers. For biopsy: all n = 216693, Hub-Met n = 218, Hub-Mic n = 110, Mic-Met n = 16 paths. For blood: all n = 1373688, Hub-Met n = 621, Hub-Mic n = 111, Mic-Met n = 102 paths, multiple testing adjustments via Benjamini-Hochberg-correction. Abbreviations: Hub - hub metabolites, Met - inflammation associated metabolites detected in metabolomics, Mic - inflammation associated metabolites detected in microbiome modelling.
