# Supplementary material for: Metabolic modeling reveals a multi-level deregulation of host-microbiome metabolic networks in IBD
Source: Nat Commun. 2025 Jun 2;16:5120. doi: 10.1038/s41467-025-60233-2 (PMC12130198; doi:10.1038/s41467-025-60233-2)
Supplement: Supplementary file 2 — Description of Additional Supplementary Files [file 41467_2025_60233_MOESM2_ESM.pdf]

File Name: Supplementary Data.xlsx
Description:
Supplementary Data 1: List of statistical parameters from linear mixed effect models associating reaction flux predicted by BacArena with disease activity score. n = 565, multiple testing adjustments via Benjamini-Hochberg-correction.

File Name: Supplementary Data.xlsx
Description:
Supplementary Data 2: List of statistical parameters from linear mixed effect models associating reaction flux predicted by MicrobiomeGS2 with disease activity score. n = 565, multiple testing adjustments via Benjamini-Hochberg-correction.

File Name: Supplementary Data.xlsx
Description:
Supplementary Data 3: List of statistical parameters from linear mixed effect models associating flux of metabolites exchanged between bacteria predicted by BacArena with disease activity score. n = 565, multiple testing adjustments via Benjamini-Hochberg-correction.

File Name: Supplementary Data.xlsx
Description:
Supplementary Data 4: List of statistical parameters from linear mixed effect models associating flux of metabolites exchanged between bacteria predicted by MicrobiomeGS2 with disease activity score. n = 565, multiple testing adjustments via Benjamini-Hochberg-correction

File Name: Supplementary Data.xlsx
Description:
Supplementary Data 5: List of statistical parameters from linear mixed effect models associating flux of metabolites exchanged with the environment predicted by BacArena with disease activity score. n = 565, multiple testing adjustments via Benjamini-Hochberg-correction.

File Name: Supplementary Data.xlsx
Description:
Supplementary Data 6: List of statistical parameters from linear mixed effect models associating flux of metabolites exchanged with the environment predicted by MicrobiomeGS2 with disease activity score. n = 565, multiple testing adjustments via Benjamini-Hochberg-correction.

File Name: Supplementary Data.xlsx
Description:
Supplementary Data 7: List of statistical parameters from linear mixed effect models associating reaction activity score, presence/absence of reactions, and flux variability results (center/range) of host models with disease activity score. n = 296 for biopsies and n = 324 for blood samples, multiple testing adjustments via Benjamini-Hochberg-correction.

File Name: Supplementary Data.xlsx
Description:

Supplementary Data 8: Detailed report of connection between microbial and host metabolism as well as blood metabolomics results.

File Name: Supplementary Data.xlsx
Description:
Supplementary Data 9: Summary of construction of different linear mixed effect models to test association of changes in metabolism to IBD phenotypes.

File Name: Supplementary Data.xlsx
Description:
Supplementary Data 10: List of statistical parameters from linear mixed effect models associating levels of blood metabolites with disease activity score. n = 150, multiple testing adjustments via Benjamini-Hochberg-correction.

File Name: Supplementary Data 11_HBMayoBiopsyNetwork.html
Description:
Supplementary Data 11: Interactive consensus network for inflammation associated host reactions in biopsy samples.

File Name: Supplementary Data 12_HBMayoBloodNetwork.html
Description:
Supplementary Data 12: Interactive consensus network for inflammation associated host reactions in blood samples.

File Name: Supplementary Data.xlsx
Description:
Supplementary Data 13: Summary of number of metabolites which are significantly changed in microbial metabolism during IBD.

File Name: Supplementary Data.xlsx
Description:
Supplementary Data 14: List of statistical parameters from paired two-sided t-test to check significant change of target metabolite flux after intervention. n = 565, multiple testing adjustments via Benjamini-Hochberg-correction.

File Name: Supplementary Data.xlsx
Description:
Supplementary Data 15: Flux values for exchange reactions in Recon3D representing the "matjes" diet used in the study.

File Name: Supplementary Data.xlsx
Description:
Supplementary Data 16: Abundance table for bacteria which could be mapped to the HRGM reference database.

File Name: Supplementary Data.xlsx

Description:
Supplementary Data 17: List taxonomic annotation and other meta data for the HRGM reference genomes detected in the study.

File Name: Supplementary Data.xlsx
Description:
Supplementary Data 18: Summary of model sizes and uptake and secretion of metabolites from microbial community models.

File Name: Supplementary Data.xlsx
Description:
Supplementary Data 19: Growth rates of bacterial models, which were excluded from the analysis because of too low predicted growth under given conditions.

File Name: Supplementary Data 20_ResponseBiopsyNetwork.html
Description:
Supplementary Data 20: Interactive consensus network for response associated host reactions in biopsy samples.

File Name: Supplementary Data 21_ResponseBloodNetwork.html
Description:
Supplementary Data 21: Interactive consensus network for response associated host reactions in blood samples.

File Name: Supplementary Data 22_RemissionBiopsyNetwork.html
Description:
Supplementary Data 22: Interactive consensus network for remission associated host reactions in biopsy samples.

File Name: Supplementary Data 23_RemissionBloodNetwork.html
Description:
Supplementary Data 23: Interactive consensus network for remission associated host reactions in blood samples.
