# Supplementary material for: Metabolic modeling reveals a multi-level deregulation of host-microbiome metabolic networks in IBD
Source: Nat Commun. 2025 Jun 2;16:5120. doi: 10.1038/s41467-025-60233-2 (PMC12130198; doi:10.1038/s41467-025-60233-2)
Supplement: Supplementary file 4 — Reporting Summary [file 41467_2025_60233_MOESM4_ESM.pdf]

nature portfolio

Corresponding author(s): Jan Taubenheim
Christoph Kaleta

Last updated by author(s): May 6, 2025

Reporting Summary

Nature Portfolio wishes to improve the reproducibility of the work that we publish. This form provides structure for consistency and transparency in reporting. For further information on Nature Portfolio policies, see our Editorial Policies and the Editorial Policy Checklist.

Statistics

For all statistical analyses, confirm that the following items are present in the figure legend, table legend, main text, or Methods section.

n/a	Confirmed	
☐	☒	The exact sample size (*n*) for each experimental group/condition, given as a discrete number and unit of measurement
☐	☒	A statement on whether measurements were taken from distinct samples or whether the same sample was measured repeatedly
☐	☒	The statistical test(s) used AND whether they are one- or two-sided<br>*Only common tests should be described solely by name; describe more complex techniques in the Methods section.*
☐	☒	A description of all covariates tested
☐	☒	A description of any assumptions or corrections, such as tests of normality and adjustment for multiple comparisons
☐	☒	A full description of the statistical parameters including central tendency (e.g. means) or other basic estimates (e.g. regression coefficient) AND variation (e.g. standard deviation) or associated estimates of uncertainty (e.g. confidence intervals)
☐	☒	For null hypothesis testing, the test statistic (e.g. *F*, *t*, *r*) with confidence intervals, effect sizes, degrees of freedom and *P* value noted<br>*Give P values as exact values whenever suitable.*
☒	☐	For Bayesian analysis, information on the choice of priors and Markov chain Monte Carlo settings
☒	☐	For hierarchical and complex designs, identification of the appropriate level for tests and full reporting of outcomes
☐	☒	Estimates of effect sizes (e.g. Cohen's *d*, Pearson's *r*), indicating how they were calculated

Our web collection on statistics for biologists contains articles on many of the points above.

Software and code

Policy information about availability of computer code

Data collection	No software was used
Data analysis	R 4.2.2: fpc 2.2-9, lme4 1.1-31, lmerTest 1.1-31, DHARMa 0.4.6, clusterProfiler 4.6.0, emmeans 1.8.2, igraph 3.4.4, ggraph 2.1.0, visNetwork 2.1.2, data.table 1.14.8, ggplot2 3.4.4, cowplot 1.1.1, RColorBrewer 1.1.3, BacArena 1.8.1, Waschina/MicrobiomeGS2 a8b022766c9600f40ebd6cc415f0f9d7104babab<br>Python 3.11.3: snakemake 7.26.0<br>Python 3.10.11: cobra 0.26.3, cobamp 0.2.1, pandas 1.3.5, numpy 1.21.6, scipy 1.7.3, troppo 0.0.6, porthmeus/corpse 0.1

For manuscripts utilizing custom algorithms or software that are central to the research but not yet described in published literature, software must be made available to editors and reviewers. We strongly encourage code deposition in a community repository (e.g. GitHub). See the Nature Portfolio guidelines for submitting code & software for further information.

Data

Policy information about availability of data

All manuscripts must include a data availability statement. This statement should provide the following information, where applicable:

- Accession codes, unique identifiers, or web links for publicly available datasets
- A description of any restrictions on data availability
- For clinical datasets or third party data, please ensure that the statement adheres to our policy

All data, analysis script, final, and intermediate results are available through Zenodo (doi: 10.5281/zenodo.13759864), scripts only are published at github (https://github.com/Porthmeus/IBDMetabolicModeling). The pipeline for context specific model reconstruction is available at github (https://github.com/Porthmeus/CSMGen_miTarget). Raw data of the sequencing was published with previous manuscripts under the GEO accession GSE191328 (Cohort 1) and GSE171770 (Cohort 2).

Research involving human participants, their data, or biological material

Policy information about studies with human participants or human data. See also policy information about sex, gender (identity/presentation), and sexual orientation and race, ethnicity and racism.

Reporting on sex and gender	We refer to patient sex with female and male, according to the sex they were born with (biological). We did not include sex specific analysis in this study for two reasons: initial checks did not indicate major differences in the different sexes for the factors of interest. Secondly, stratification of the data would have led to loss in statistical power.
Reporting on race, ethnicity, or other socially relevant groupings	Does not apply
Population characteristics	Does not apply
Recruitment	Does not apply
Ethics oversight	Data was approved by the ethics committee of the Medical Faculty at Kiel University (A156/03, A124/14)

Note that full information on the approval of the study protocol must also be provided in the manuscript.

Field-specific reporting

Please select the one below that is the best fit for your research. If you are not sure, read the appropriate sections before making your selection.

☒ Life sciences ☐ Behavioural & social sciences ☐ Ecological, evolutionary & environmental sciences

For a reference copy of the document with all sections, see nature.com/documents/nr-reporting-summary-flat.pdf

Life sciences study design

All studies must disclose on these points even when the disclosure is negative.

Sample size	Sample size was chosen in previous clinical trials, we used the data and reanalyzed new aspects of it. Initial sample size was chosen by statistical power analysis to evaluate clinical outcome of different IBD intervention therapy.
Data exclusions	Data was excluded based on the quality of sequencing.
Replication	All code was run with small test datasets and manual evaluation of correct results before applying to the complete datasets.
Randomization	Does not apply, because all patients included were diagnosed with active IBD at begin of study. Treatment outcome over time determined grouping of the patients.
Blinding	Blinding was not relevant, as it is a reanalysis of data taken in a different context

Reporting for specific materials, systems and methods

We require information from authors about some types of materials, experimental systems and methods used in many studies. Here, indicate whether each material, system or method listed is relevant to your study. If you are not sure if a list item applies to your research, read the appropriate section before selecting a response.

Materials & experimental systems

n/a	Involved in the study	
☒	☐	Antibodies
☒	☐	Eukaryotic cell lines
☒	☐	Palaeontology and archaeology
☒	☐	Animals and other organisms
☐	☒	Clinical data
☒	☐	Dual use research of concern
☒	☐	Plants

Methods

n/a	Involved in the study	
☒	☐	ChIP-seq
☒	☐	Flow cytometry
☒	☐	MRI-based neuroimaging

Clinical data

Policy information about clinical studies

All manuscripts should comply with the ICMJEguidelines for publication of clinical research and a completedCONSORT checklist must be included with all submissions.

Clinical trial registration	EudraCT number 2016-000205-36 and ClinicalTrials.gov NCT02694588
Study protocol	Please refer to the original publications for the clinical trials: https://doi.org/10.1053/j.gastro.2021.02.062 and https://doi.org/10.1136/gutjnl-2018-316023
Data collection	Please refer to the original publications for the clinical trials: https://doi.org/10.1053/j.gastro.2021.02.062 and https://doi.org/10.1136/gutjnl-2018-316023
Outcomes	Please refer to the original publications for the clinical trials: https://doi.org/10.1053/j.gastro.2021.02.062 and https://doi.org/10.1136/gutjnl-2018-316023

Plants

Seed stocks	*Report on the source of all seed stocks or other plant material used. If applicable, state the seed stock centre and catalogue number. If plant specimens were collected from the field, describe the collection location, date and sampling procedures.*
Novel plant genotypes	*Describe the methods by which all novel plant genotypes were produced. This includes those generated by transgenic approaches, gene editing, chemical/radiation-based mutagenesis and hybridization. For transgenic lines, describe the transformation method, the number of independent lines analyzed and the generation upon which experiments were performed. For gene-edited lines, describe the editor used, the endogenous sequence targeted for editing, the targeting guide RNA sequence (if applicable) and how the editor was applied.*
Authentication	*Describe any authentication procedures for each seed stock used or novel genotype generated. Describe any experiments used to assess the effect of a mutation and, where applicable, how potential secondary effects (e.g. second site T-DNA insertions, mosiacism, off-target gene editing) were examined.*
